# Targeting the vasculature in cardiometabolic disease

**DOI:** 10.1172/JCI148556

**Published:** 2022-03-15

**Authors:** Nabil E. Boutagy, Abhishek K. Singh, William C. Sessa

**Affiliations:** Vascular Biology and Therapeutics Program and Department of Pharmacology, Yale University School of Medicine, New Haven, Connecticut, USA.

## Abstract

Obesity has reached epidemic proportions and is a major contributor to insulin resistance (IR) and type 2 diabetes (T2D). Importantly, IR and T2D substantially increase the risk of cardiovascular (CV) disease. Although there are successful approaches to maintain glycemic control, there continue to be increased CV morbidity and mortality associated with metabolic disease. Therefore, there is an urgent need to understand the cellular and molecular processes that underlie cardiometabolic changes that occur during obesity so that optimal medical therapies can be designed to attenuate or prevent the sequelae of this disease. The vascular endothelium is in constant contact with the circulating milieu; thus, it is not surprising that obesity-driven elevations in lipids, glucose, and proinflammatory mediators induce endothelial dysfunction, vascular inflammation, and vascular remodeling in all segments of the vasculature. As cardiometabolic disease progresses, so do pathological changes in the entire vascular network, which can feed forward to exacerbate disease progression. Recent cellular and molecular data have implicated the vasculature as an initiating and instigating factor in the development of several cardiometabolic diseases. This Review discusses these findings in the context of atherosclerosis, IR and T2D, and heart failure with preserved ejection fraction. In addition, novel strategies to therapeutically target the vasculature to lessen cardiometabolic disease burden are introduced.

## Introduction

Excess calorie consumption and reduced exercise have led to a growing number of individuals who are overweight or obese, with an estimated prevalence of approximately 65% of US adults and over 1.5 billion people worldwide (1). Obesity is associated with substantial health care burden, with an estimated annual medical cost of $210 billion (2). Excess weight gain is accompanied by ectopic fat deposition in extra-adipose tissue that contributes to the development of hyperlipidemia, insulin resistance (IR), and type 2 diabetes (T2D) (3, 4). This disease constellation, in conjunction with additional cardiovascular (CV) risk factors, significantly increases the incidence of CV disease (CVD), such as myocardial infarction and heart failure (HF). Thus, understanding the cellular and molecular processes that underlie cardiometabolic changes that occur during obesity is necessary for the design of optimal medical therapies to attenuate or prevent the sequelae of this disease.

### Endothelium as an arbiter of homeostasis versus disease.

The vascular endothelium is a single layer of cells lining the entire interior surface of all vascular networks. Under normal conditions, the healthy endothelium controls vascular homeostasis by modulating vascular tone, maintaining blood fluidity and flow, controlling vessel wall permeability, and counteracting vascular inflammation (5). As such, endothelial dysfunction, or a failure of the endothelium to perform any of these critical functions, is essential in the initiation and progression of atherosclerosis and metabolic disease (6). Numerous reports demonstrated that dyslipidemia and hyperglycemia result in endothelial dysfunction (7–12). In addition, the release of proinflammatory cytokines (IL-6, TNF-α; refs. 5, 13) and adipokines (resistin; ref. 14) from inflamed adipose tissue (AT) can perturb endothelial function (15). Thus, endothelial dysfunction represents a pathophysiological link between metabolic and CV disease, and recent discoveries have uncovered an important role of the capillary endothelium and microvasculature in regulating metabolic functions under homeostatic and pathological conditions (16). As cardiometabolic disease progresses, so do vascular inflammation, remodeling, and dysfunction in all segments of the vasculature, including large conduit arteries, resistance vessels (arterioles), capillaries, and conductance vessels (veins and lymphatics). These changes can exacerbate cardiometabolic disease progression. Thus, this Review’s goal is to highlight recent cellular and molecular data that implicate the vasculature as an initiating and instigating factor in atherosclerosis, IR, and HF development.

## Endothelial cells, lipoprotein processing, and atherosclerosis

During the fed state, intestine-derived apolipoprotein B-48–containing (apoB48-containing) chylomicrons are the primary source of dietary lipids. Conversely, during the fasting state, liver-derived apoB100-rich VLDL particles carry endogenous lipids to peripheral tissues (17). Chylomicrons and VLDL are triglyceride-rich lipoprotein (TRL) particles that distribute energy-rich free fatty acids (FFAs) to metabolically active tissues including the heart, AT, and skeletal muscle. Liberation of FFAs from TRLs occurs via the interaction of TRLs with lipoprotein lipase (LPL) present on the luminal surface of microvascular capillary endothelial cells (ECs) (18). LPL is transported from subendothelial space to the luminal surface of ECs through its interaction with the capillary EC protein glycosylphosphatidylinositol-anchored HDL-binding protein 1 (GPIHBP1). GPIHBP1 is essential in anchoring LPL on the surface of capillaries during intravascular lipolysis of triglycerides (TGs) into FFAs and glycerol. Following lipolysis, FFAs can traverse the endothelium passively (flip-flop mechanism), through vectorial transport, or via receptor-mediated uptake. Many of these pathways of lipoprotein metabolism and FFA uptake mechanisms in ECs were recently reviewed in detail (19, 20).

The influx of TRL-derived FFAs in metabolic tissues appears within minutes after injection of labeled chylomicron/VLDL-TG and is largely dependent on LPL activity (21, 22); loss-of-function GPIHBP1 or LPL mutations lead to severe hypertriglyceridemia (23–25). LPL activity is negatively regulated by angiopoietin-like proteins (ANGPTL3/4/8) (26–29). ANGPTLs are secretory proteins that inhibit LPL directly by catalyzing the dissociation of catalytically active LPL homodimers into inactive monomers (30). These proteins are highly expressed in metabolic tissues and have recently emerged as modulators of LPL-mediated lipid partitioning under differing nutritional states. Genome-wide association, exome sequencing, and DiscovEHR human genetic studies associate loss-of-function mutations in ANGPTL3 or ANGPTL4 with lower plasma TGs and LDL, lower fasting blood glucose, increased insulin sensitivity, and reduced CVD incidence (31–36). Despite being secreted proteins, ANGPTL3 and ANGPTL4’s effects appear to be tissue specific. ANGPTL3 is exclusively expressed in the human and mouse liver, and ANGPTL3 deletion in mice leads to increased post-heparin plasma LPL and endothelial lipase activity, reduced levels of circulating TGs and LDL, and reduced atherosclerosis (37–40). ANGPTL4 is expressed and secreted from AT (both white and brown) and rodent and primate liver (32, 41–43). Recent studies showed that ANGPTL4 deficiency in either adipocytes (Ad-KO mice) or hepatocytes (Hmut [hepatocyte mutant] mice) lowers plasma TG and cholesterol levels by increasing LPL or hepatic lipase activity, respectively (43, 44). As a result, Ad-KO and Hmut mice are protected from atherosclerosis during hyperlipidemic conditions, despite no observable changes in systemic inflammation. In addition, Hmut mice have improved glucose tolerance, insulin sensitivity, and reduced adiposity during high-fat diet–induced (HFD-induced) obesity. Together, these studies indicate that targeting ANGPTL4 in a tissue-specific manner can improve cardiometabolic health in rodent models of atherosclerosis and T2D. Although ANGPTL4 is expressed in ECs and is induced by hypoxia, the EC-specific role of ANGPTL4 in TG partitioning and atherosclerosis has yet to be studied.

### Chylomicron remnants and atherosclerosis.

After peripheral lipolysis, a major portion of remnant chylomicrons still contain substantial amounts of lipid. Chylomicron remnants found in human atheromas may contribute to atherosclerosis through several mechanisms (45–47). First, studies have reported that completely lipolyzed chylomicron remnants (~50 nm diameter) can contain approximately 40-fold greater cholesterol than LDL (48, 49). Second, a recent study revealed that the aorta of LPL-deficient mice can take up chylomicron remnants via a receptor-mediated pathway and transfer FFAs from endocytosed chylomicrons to lipid droplets in ECs (50). These findings are interesting, as the aortic endothelium has negligible levels of GPIHBP1 and low levels of LPL. Although provocative, the role of chylomicron remnant particles in atherosclerosis requires further study.

### Endothelium-mediated LDL uptake and atherosclerosis progression.

It is appreciated that LDL uptake, entry, and retention in the vessel wall are a key initiating factor in development of atherosclerosis (51). Early studies demonstrated that LDL’s passage from blood into and across the endothelium occurs via an LDL receptor–independent mechanism and may involve non-clathrin-coated vesicles, namely caveolae (52). Interestingly, genetic loss of the caveolae coat protein, caveolin-1 (CAV-1), reduces LDL uptake into ECs and retards atherosclerosis in the presence of elevated TG and cholesterol levels (53, 54). Moreover, recent investigation has begun to uncover novel mechanisms of receptor-mediated LDL transcytosis during the initiation and progression of atherosclerosis (55).

A study combining genome-wide siRNA screening with high-content confocal imaging identified activin A receptor–like type 1 (ALK1) as a novel low-affinity, high-capacity receptor for LDL particles in ECs that promote LDL transcytosis during hyperlipidemic conditions (56). Accordingly, EC-specific ALK1 depletion in mice reduced LDL uptake into vessels in vivo. More recently, the high-affinity HDL-binding receptor scavenger receptor class B type 1 (SR-B1) was identified as a novel player in LDL transcytosis in the aortic endothelium, and EC-specific deficiency of SR-B1 reduces atherosclerosis in atheroprone mice (57, 58). Interestingly, ALK1 (59) and SR-B1 have been localized in caveolae, suggesting that ALK1, SR-B1, and CAV-1 may mediate LDL transcytosis across endothelium in a complementary way. Indeed, future studies and therapeutic strategies are needed to test this supposition as a novel mechanism to treat atherosclerosis in conjunction with lipid-lowering approaches.

### ApoM at the interface of lipoprotein metabolism and vascular function.

ApoM is synthesized in the liver and binds primarily to HDL and to a lesser extent to VLDL, LDL, and chylomicrons (60). ApoM-containing HDL is atheroprotective and promotes cholesterol efflux from macrophages and HDL formation (61). Recent studies have shown that apoM/HDL serves as a major molecular chaperone in plasma for the biologically active lipid sphingosine-1-phosphate (S1P) (62). S1P promotes EC barrier function and endothelial nitric oxide synthase (eNOS) activation and protects against inflammation and atherosclerosis, and many of these actions are mediated physiologically by the apoM/S1P complex (63). In addition to its vascular actions, apoM/S1P also regulates insulin sensitivity and TG metabolism in AT, as discussed elsewhere in detail (63, 64).

**Figure 1:**
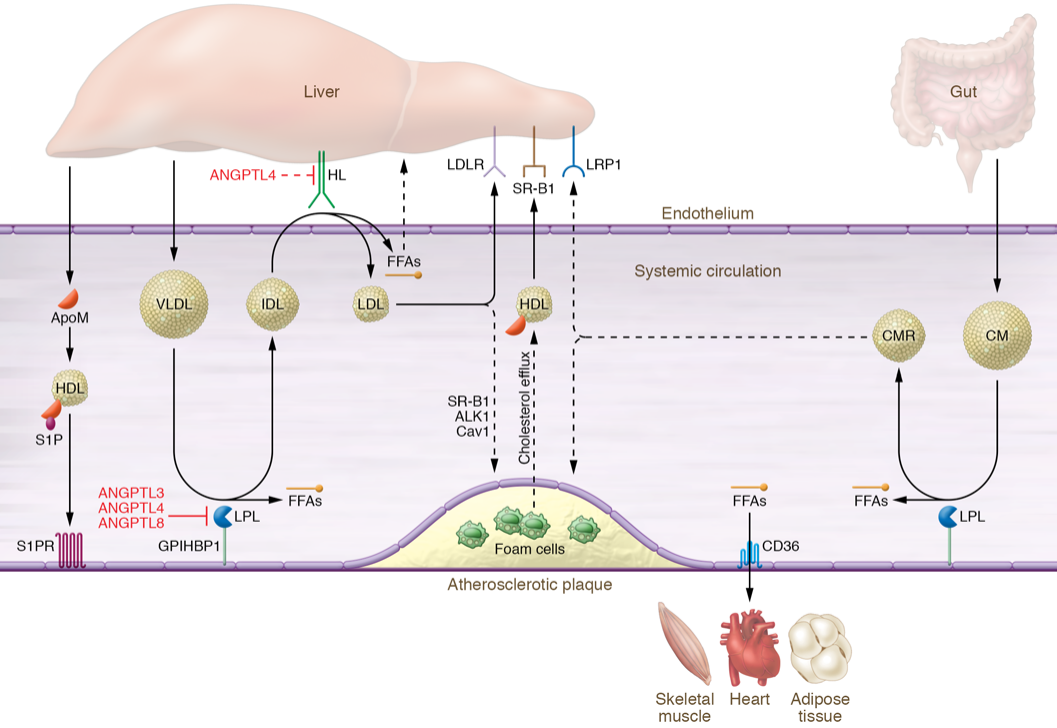
Schematic overview of systemic lipoprotein metabolism. Triglyceride-rich lipoproteins such as VLDL and chylomicron (CM) are produced by the liver and gut, respectively, which distribute FFAs to various metabolic tissue(s) like muscle, heart, and adipose by interacting with the GPIHBP1-bound endothelial LPL enzyme. LPL activity is regulated by secreted ANGPTL3, ANGPTL4, and ANGPTL8. In subsequent peripheral lipolysis, VLDL and CM are converted into intermediate-density lipoprotein (IDL) and chylomicron remnants (CMRs). In the liver, HL liberates FFAs from IDL and converts them into LDL particles. HL activity is inhibited by ANGPTL4. The liver clears a large portion of remnants (LDL and CMRs) via hepatic receptors (LDLR and LRP1). Under hyperlipidemic conditions, some fractions of LDL or CMRs accumulate and oxidize in the subendothelial space of a large artery, which is subsequently taken up by macrophages that develop into foam cells within atherosclerotic plaques. Liver-derived Apo M complexed with sphingosine-1-phosphate (S1P) on HDL may modulate atherosclerotic plaque progression, first by interacting with endothelial S1P receptors (S1PRs) to maintain vascular integrity and suppress inflammation (lower left), and second, by reducing cholesterol overload of macrophages through the promotion of cholesterol efflux (center of figure).

Figure 1 summarizes the major findings regarding the relationship between EC lipoprotein processing and atherosclerosis to date.

## Current and future approaches to address additional CV risk

Statin therapy combined with other cholesterol-lowering strategies is the mainstay in reducing risk of atherosclerosis and its sequelae. However, some individuals still demonstrate a high risk of atherosclerotic incidents despite low to moderate levels of LDL cholesterol. Thus, “residual risk” in these patients implies that mechanisms beyond cholesterol are important in atherosclerosis disease progression (65). Epidemiologically, TRLs are strong independent predictors of atherosclerotic CVD (66). Consistently, lowering circulating TG levels with icosapent ethyl (purified eicosapentaenoic acid [EPA]) in patients on a maximally tolerated dose of statins significantly reduced risk of CV events/death by 25% compared with placebo in CVD patients or patients with diabetes who had elevated fasting TGs (135–499 mg/dL) and one additional risk factor (67). These results provide supporting evidence that perturbed TG production and clearance, as is common in obesity and T2D, increase CVD susceptibility. However, these results may be unique to purified icosapent, as other large studies do not support use of mixed omega-3 carboxylic acids for reducing major CV events in high-risk patients (68). In addition to its TG-lowering actions, EPA improves flow-mediated dilation (an index of endothelial function) by reducing inflammation and enhancing NO bioavailability in patients with mild hypertriglyceridemia (69).

Considering the role of the microvasculature in mediating TG clearance, pharmacological targets that promote vascular partitioning of TGs have been explored in preclinical models of cardiometabolic disease and in clinical trials (70–73). Along these lines, a recent phase II clinical trial demonstrated that an antisense oligonucleotide targeting hepatic ANGPTL3 (vupanorsen) led to reduction in circulating TGs and LDL in healthy and T2D individuals (71). In addition, the FDA recently approved evinacumab (trade name Evkeeza), a monoclonal antibody against human ANGPTL3, following phase III clinical trial results demonstrating impressive (49%) reduction in circulating LDL in patients with severe, inherited forms of hypercholesterolemia (72, 73). Although exciting, whether targeting of ANGPTL3 to enhance TRL clearance can reduce CV events in patients on lipid-lowering therapy with atherosclerotic CVD remains undetermined.

## Role of the microvasculature in metabolic homeostasis

In addition to indirect effects of EC processing of TRL, emerging evidence suggests that the microvasculature has a direct role in maintaining the function of highly metabolic organs under normal physiological conditions. Importantly, disruption of microvascular function has been implicated in disease progression of obesity-induced IR and T2D. Below, we summarize recent studies supporting these concepts.

### The vasculature and adipose tissue expansion.

The relationship between the vasculature network and AT under normal physiology and diet-induced obesity has been well described (74, 75). Briefly, AT is highly vascularized with a dense capillary network that is essential for tissue homeostasis. During adipogenesis, the vasculature can deliver stem cells to AT as well as mobilize adipocyte progenitor cells and preadipocytes from mural and perivascular compartments, respectively (76–78). Therefore, the vasculature supports AT homeostasis and directly contributes to AT expansion.

During initial stages of energy surplus, AT expands to store excess energy in the form of TGs. Physiological or “healthy” AT expansion is a coordinated response that involves remodeling of extracellular matrix (ECM) and angiogenesis to allow for adequate adipocyte expansion and maintain oxygen/nutrient delivery, respectively. Local hypoxia is a potent stimulus for angiogenesis leading to release of several proangiogenic factors from adipocytes through the action of HIF1α (79, 80). Of the angiogenic factors released from adipocytes, VEGFA/VEGFR2 signaling accounts for the most angiogenic activity (81). Endothelial VEGFA/VEGFR2 signaling, through paracrine action on progenitor cells, is also important in “beiging” of superficial white AT (WAT) to resemble more metabolically active brown AT (BAT) in rodent models of obesity (82). HIF1α signaling was also implicated in the activation of local immune cells, as well as the recruitment of circulating immune cells to AT (80). Importantly, macrophages facilitate ECM remodeling, removal of apoptotic or necrotic cells, and VEGFA secretion to further support angiogenesis. Under states of chronic energy surplus, AT expansion becomes pathological and is characterized by impaired angiogenesis, unresolved inflammation, tissue fibrosis, and an accumulation of necrotic/apoptotic cells. In addition, inflamed AT is insulin resistant, and thus exhibits elevated levels of intracellular lipolysis during the fasting and fed states that contribute to ectopic lipid deposition, inflammation, and IR in other tissues.

Considering that angiogenesis is essential for AT expansion, targeting of neovascularization at different stages of diet-induced obesity has been explored. Several early studies demonstrated that inhibiting angiogenesis in AT resulted in AT regression and improvements in metabolic function in rodent models of advanced obesity (83, 84). Recent studies challenge this, since physiological angiogenesis during the progression of diet-induced obesity protects AT from hypoxia and inflammation, ectopic lipid deposition, and systemic IR (81). Most studies (85, 86), but not all (87), support these latter findings. Specifically, deleting *Vegfa* from adipocytes promoted poor AT vascularization during high-fat feeding in mice and was associated with AT inflammation and worse systemic IR in comparison with wild-type littermates (86). Reciprocally, VEGFA overexpression in both BAT and WAT rendered protection against diet-induced AT inflammation and systemic metabolic derangements (81, 85, 86). More recently, adenoviral gene delivery of *Vegfb* was demonstrated to attenuate AT inflammation and prevent systemic IR without influencing body weight in mice fed a HFD (88). More interestingly, delayed *Vegfb* gene delivery impeded progression of metabolic dysfunction in mice exposed to HFD for 2 months before gene delivery. Mechanistically, VEGFB’s beneficial effects occur through its interaction with the decoy receptor VEGFR1, thus improving VEGFA’s binding affinity for VEGFR2. Considering dichotomous outcomes depending on the timing of intervention, translation of preclinical findings to humans will be challenging, especially since patients with IR are often already overweight or obese.

### Endothelium-derived NO, blood flow, and metabolism.

The endothelium elegantly balances the release of vasodilatory and vasoconstrictive substances to allow for tight regulation of vascular resistance, and thus tissue perfusion. The most well characterized of these factors is endothelium-derived NO, which promotes cGMP signaling, resulting in dilation of vascular smooth muscle in resistance vessels (89). Logically, blood flow mediates oxygen and nutrient delivery to metabolic tissues at rest, during exercise, and during the postprandial period (90, 91). Along these lines, eNOS-KO mice, deficient in the main enzyme generating circulating NO, display impaired skeletal muscle glucose and FFA uptake during an acute exercise bout, which is directly related to reduction in tissue blood perfusion (90). Interestingly, insulin signaling via endothelial insulin receptors (INSRs) leads to eNOS activation and subsequent recruitment of skeletal muscle capillaries during the postprandial period, which contributes to insulin and glucose delivery to skeletal muscle (92). Several studies have demonstrated impairments in endothelial insulin signaling during high-fat feeding in rodents (93–95). Interestingly, endothelial IR may occur prior to systemic IR in mice exposed to a HFD (93), and accumulating evidence suggests that perturbations in endothelial insulin signaling can directly contribute to whole-body IR (92, 96). Mechanistically, some groups have argued that impairments in skeletal muscle capillary recruitment secondary to reduced eNOS activity are largely responsible for this relationship, while others have proposed impaired insulin delivery through the nonfenestrated endothelium to underlying parenchyma cells as an alternative mechanism (see below).

In addition to regulating blood flow, NO can have both short- and long-term effects on systemic metabolism. For example, NO/cGMP signaling contributes to skeletal (97) and heart (98) muscle glucose uptake through partial activation of AMP-activated protein kinase (AMPK), which then leads to glucose transporter 4 (GLUT-4) translocation to the cellular membrane of myocytes. Interestingly, in ECs, AMPK activation was demonstrated to activate eNOS (99, 100), thus supporting a bidirectional relationship between energy sensing and NO signaling. In addition, NO can toggle the enzymatic activity of key metabolic proteins through posttranslational mechanisms. Specifically, cysteine-*S*-nitrosylation at Cys^238^ in liver very long acyl-CoA dehydrogenase (VLCAD), a crucial enzyme in β-oxidation of fatty acids, improves its catalytic efficiency (101). Furthermore, NO was demonstrated to exert long-term effects on energy homeostasis through the regulation of key energy sensors and transcription factors. In skeletal muscle, together with AMPK, NO/cGMP signaling increases the activity of PPARγ coactivator 1α (PGC1α) (102), a transcription factor that stimulates mitochondrial biogenesis and oxidative metabolism (103). Consistently, eNOS-KO mice have defective skeletal muscle β-oxidation (104) and reduced activity of several key enzymes involved in oxidative metabolism (90). In AT, endothelium-derived NO was demonstrated to stimulate PPARα and -γ gene transcription, which was associated with increased metabolic activity and differentiation (105). As follows, eNOS overexpression protects mice from weight gain during high-fat feeding (105). However, due to the complexities of NO biology, the translational aspect of these studies is challenging (see below).

### Transendothelial insulin transport.

Along with blood flow and tissue perfusion, transendothelial transport (TET) of insulin contributes to insulin delivery to underlying parenchymal cells in tissue where nonfenestrated endothelial networks exist. Notably, intact INSR signaling appears to be of paramount importance for mediating TET in both cell culture and animal models. In vitro, inhibiting downstream components of the insulin signaling pathway dramatically impairs insulin uptake in aortic ECs (106). In rodent models, endothelium-specific INSR deletion slows the kinetics of insulin delivery to underlying peripheral tissue in certain areas of the brain, which predisposes animals to overeating and systemic IR when they are challenged with HFD (96). Interestingly, evidence suggests that CAV-1 is involved in apical-to-basolateral transport of insulin, and this process is partially dependent on intact insulin signaling (107). A recent study demonstrated that endothelial NOTCH signaling reduces CAV-1 levels as well as other proteins involved in caveolae formation during high-fat feeding (108). In this study, endothelium-specific NOTCH deletion rendered protection from HFD-induced IR in skeletal muscle and AT. Interestingly, CAV-1 was demonstrated to stabilize INSRs in AT (109); thus, caveolae formation may not be necessary for insulin transport in the endothelium. In addition to receptor-mediated processes, others have proposed that insulin TET occurs via a nonsaturable, INSR-independent fluid-phase process (110, 111). Indeed, it is possible that both receptor-dependent and -independent mechanisms are operative in vivo, as endothelium-specific INSR deletion only delays insulin signaling kinetics, rather than eliciting complete loss of insulin responsiveness in parenchymal tissue following insulin injection (96).

### Endothelium-mediated fatty acid uptake.

As mentioned above, FFAs can be liberated from TRLs via local endothelial LPL activity. In addition, lipolysis of adipocyte TGs leads to elevated circulating FFAs bound to albumin. As with insulin delivery, FFAs must traverse the endothelium before delivery to underlying parenchymal cells in tissues with nonfenestrated capillaries. Once believed to be a completely passive process, recent in vivo evidence has bolstered the idea that active/facilitated endothelial transport is also operative in delivery of FFAs to underlying parenchymal cells. For example, mice with endothelium-specific CD36 deletion have reduced tissue FFA uptake during fasting, which in turn renders protection against HFD-induced glucose intolerance and IR in comparison with control littermate mice (112). Alternatively, studies using NanoSIMS technology (CAMECA) to visualize the uptake of chylomicron-derived FFAs across capillaries demonstrated an extremely rapid uptake process that was not influenced by CD36 deficiency (22). At first glance, these studies seem contradictory, but they support early observations that LPL-mediated VLDL hydrolysis or albumin-bound FFA fractions utilize CD36 for FFA uptake, whereas chylomicron hydrolysis and subsequent FFA transport to cardiac tissue are not mediated by CD36 (113). The mechanisms determining the route of FFA entry are unclear, but may arise from differences in receptor affinities, concentrations of FFA in its various bound states, or the receptor density of other EC fatty acid–binding proteins (FABPs). Along these lines, the fatty acid chaperone proteins FABP4 and FABP5 are abundantly expressed in the capillary endothelium of cardiac and skeletal muscle, and deleting or downregulating these proteins impairs cardiac and skeletal muscle FFA uptake (114, 115). As the heart largely relies on FFAs to meet its large energetic demands, impairing endothelium-mediated cardiac FFA transport/uptake has been associated with cardiac hypertrophy and dysfunction (115, 116). Beyond chaperone proteins, recent work has demonstrated that fatty acid transport protein 4 (FATP4) is localized to the endoplasmic reticulum in ECs and mediates cellular uptake of FFAs via acyl-CoA synthetase (ACS) activity and mitochondrial ATP production (117). These studies align with the hypothesis of vectorial transport (118) (or acylation) in that FFA “activation” via the ATP-dependent covalent addition of a CoA group by ATP-dependent ACS proteins decreases the intracellular FFA concentration and drives FFA uptake by the law of mass action. FATP3 and other acyl-CoA synthetases (acyl-CoA synthetase long chain family member 1 [ACSL1]) expressed in capillary ECs likely mediate FFA uptake via similar mechanisms (117).

Early studies demonstrated that PPARγ regulates an EC transcriptional program (FABP4, CD36, and GPIHBP1) that largely dictates tissue uptake and clearance of FFAs during fasting and the postprandial period (119). More interesting was the fact that endothelium-specific PPARγ-KO mice were protected from HFD-induced IR, despite increased liver adiposity. Other studies show that the transcription factors mesenchyme homeobox 2 (Meox2) and transcription factor 15 (Tcf15) regulate the expression of CD36, FATBP4, FATBP5, and GPIHBP1, but not FATP3 and FATP4, in cardiac capillary ECs (115). As expected, partial deletion of Meox2 and Tcf15 reduced cardiac FFA uptake and promoted cardiac dysfunction and fibrosis in mice. Similarly, disrupting NOTCH signaling in ECs severely blunted cardiac uptake of FFAs secondary to downregulation of endothelial lipase, FABP4, FABP5, and CD36, while increasing the expression of the LPL inhibitor ANGPTL4 (116). The reduction in cardiac FFA uptake led to a compensatory increase in glucose uptake, cardiac hypertrophy, and a dramatic reduction in systolic performance.

Although FFA uptake through different pathways is well described, the fate of FFAs once internalized is less clear. Recent data showed that ECs contain the machinery to transiently form and turn over lipid droplets (LDs) in response to FFA loading in both micro- and macrovascular vessels (120). In microvascular ECs, LD synthesis buffers lipotoxicity mainly through the actions of diacylglycerol *O-*acyltransferase 1 (DGAT1). Alternatively, LD hydrolysis, which is rate-limited by adipose triglyceride lipase (ATGL), provides FFAs for mitochondrial oxidation or delivery to parenchymal cells (120). Interestingly, EC lipolysis of LDs is negatively regulated by CAV-1 in microvessels (121). To date, the function of LD metabolism in large-vessel ECs remains unclear. Global ATGL loss can trigger EC dysfunction in large vessels (122), suggesting that LD turnover may also be important in large-vessel EC homeostasis.

Interestingly, hormone peptides (apelin [ref. 123]), growth factors (VEGFB [ref. 124], angiopoietin-2 [ref. 125]), and metabolites (3-hydroxybutyrate [ref. 127]) released from metabolic tissues can alter the expression level or localization of several FFA transporters, demonstrating coordinated regulation between the endothelium, the circulating milieu, and the underlying parenchymal cells. For example, 3-hydroxyisobutyrate (3-HIB) is a metabolite released from excessive branched-chain amino acid metabolism in skeletal muscle of genetically diabetic mice. In a paracrine manner, 3-HIB promotes transendothelial FFA transport via activation of FATP4, and consequently contributes to muscle lipid accumulation and IR in skeletal muscle (126). Conversely, angiopoietin-2 released from superficial AT (SAT) during nutrient overload prevents ectopic lipid accumulation in other insulin-sensitive tissues by activating endothelial α_5_β_1_ integrin signaling to enhance FFA uptake in SAT. Notably, several of these factors have other physiological functions besides influencing EC-mediated FFA uptake, such as the role of VEGFB in AT angiogenesis (88). Thus, targeting these factors may not be therapeutically efficacious in comorbid disease states, and deeper molecular understanding of signaling events that dictate aberrant EC FFA delivery in a tissue- and disease-specific context is needed.

**Figure 2:**
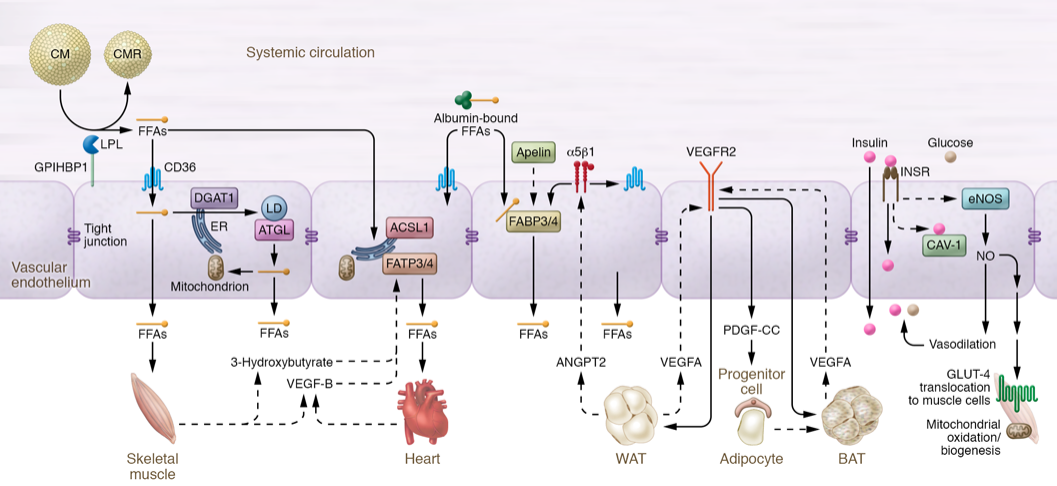
Schematic overview of endothelial microvasculature in fatty acid uptake and systemic metabolism. FFAs, either bound to albumin, or derived from LPL-mediated hydrolysis of circulating TRLs, traverse the endothelium by passive diffusion (flip-flop), receptor-mediated uptake (via CD36), and/or vectorial transport (via FATP4). FATP3 and ACSL1 and other acyl-CoA synthases likely facilitate vectorial transport, and mitochondrial ATP also appears to be important. ECs contain machinery to esterify FFAs into LDs, a DGAT1-dependent process that may protect ECs from ER stress. Conversely, LDs may be hydrolyzed, a largely ATGL-dependent process that liberates FFAs for mitochondrial oxidation and/or parenchymal delivery. Circulating bioactive molecules such as apelin can regulate FABP4 transcription, and bioactive molecules released from muscle cells, including 3-HIB and VEGFB, can regulate FATP3 and FATP4 expression. In addition, adipose tissue–derived ANGPT2 can activate endothelial α5β1 signaling to trigger FFA transport via CD36 and FATP3 into superficial adipose tissue. Adipose tissue–derived VEGFA signals via EC VEGFR2 to support both WAT and BAT angiogenesis and also plays a role in WAT “beiging” via EC VEGFR2 signaling and PDGF-CC release, which in turn activates preadipocytes to transform into BAT. In addition to FFA uptake, ECs participate in systemic metabolism. EC INSR signaling mediates insulin delivery to parenchymal cells via phosphorylation and activation of eNOS, leading to the production of NO, a potent vasodilator. INSR signaling also cooperates with CAV-1 to facilitate insulin TET via caveolae-dependent and -independent mechanisms. Insulin TET may also occur via a nonsaturable, INSR-independent fluid-phase process. In addition to insulin TET, NO increases blood flow and tissue perfusion to facilitate glucose and FFA delivery to parenchymal cells. Beyond blood flow, NO can partially activate AMPK via cGMP signaling, leading to GLUT-4 translocation to the plasma membranes of muscle cells. Moreover, via cysteine-*S*-nitrosylation reactions, NO modulates several key metabolic enzymes. Last, NO/cGMP signaling participates in the long-term regulation of mitochondrial metabolism via interactions with AMPK and PGC1α, a master transcriptional regulator of mitochondrial biogenesis and oxidative metabolism.

Figure 2 summarizes the major findings of the role of the microvasculature in mediating metabolic homeostasis.

## The vasculature and HF with preserved ejection fraction

HF is a major public health problem affecting approximately 6 million people in the United States. Community-based studies indicate that roughly 50% of HF patients present with the clinical diagnosis of HF with preserved ejection fraction (HFpEF), while the remaining patient population present with HF with reduced ejection fraction (HFrEF) (127, 128). While clinical symptoms and mortality are similar among patients with HFpEF and HFrEF, these HF phenotypes display pronounced differences in patient demographics and underlying pathophysiology of left ventricular (LV) remodeling (129, 130). Specifically, myocardial infarction is less common in HFpEF compared with HFrEF, whereas older age, female sex, hypertension, obesity, and T2D are more common in HFpEF (131). In terms of cardiac structure/function, HFpEF is characterized by diastolic dysfunction, LV hypertrophy, myocyte stiffening, fibrosis, and microvascular dysfunction/rarefaction (131). Despite the increase in HFpEF incidence (132), there are limited data to support effective therapies for metabolically driven HF, partly because of a lack of mechanistic understanding of the cellular and molecular pathways that underpin this complex clinical syndrome.

### The microvasculature in HFpEF.

Myocardial perfusion is largely dictated by coronary vascular resistance at the level of the microvasculature (133). Thus, microvascular dysfunction can lead to perfusion-demand mismatch and endocardial ischemia, even in the presence of angiographically normal coronary arteries (134). Invasive studies indicate that up to 80% of HFpEF patients have coronary microvascular dysfunction (135). Importantly, endocardial ischemia due to microvascular dysfunction can impair both active and passive relaxation of the ventricle during diastole. Specifically, myocyte relaxation is an energy-dependent process involving cytosolic calcium uptake by sarcoplasmic/endoplasmic reticulum calcium-ATPase (SERCA); thus a reduction in myocardial perfusion can impair this phase of diastole (136). On the other hand, chronic ischemia is associated with fibrosis development and increased passive stiffness of the heart (137, 138). Several studies have demonstrated that coronary microvascular dysfunction is associated with lower diastolic relaxation velocities and elevated filling pressures in HFpEF patients, either at rest or during exertion (139–142). In addition, the magnitude of cardiac fibrosis in HFpEF patients is associated with impaired diastolic relaxation (143), and some studies show that microvascular rarefaction is directly associated with cardiac fibrosis in these patients (138).

Epidemiologically, circulating inflammatory cytokines, obesity, and T2D portend greater risk of developing incident HFpEF than HFrEF (144, 145). Endomyocardial tissue biopsy samples from HFpEF patients show evidence of microvascular inflammation/oxidative stress, as well as increased numbers of immune cell infiltrates (CD3^+^, CD11a^+^, CD45^+^) (146, 147). Since microvascular disease is present in most HFpEF patients (135), impacts LV dysfunction/remodeling (142, 148, 149), and independently predicts all-cause mortality (140), it has been hypothesized that microvascular dysfunction caused by systemic inflammation mediates pathophysiological cardiac remodeling in HFpEF (150, 151).

Beyond initiating immune cell recruitment to the vasculature, inflammatory cytokines contribute to endothelial dysfunction by creating an imbalance between antiinflammatory/vasodilatory factors (i.e., decreased NO) and inflammatory/vasoconstrictive factors (i.e., increased endothelin-1) (152, 153). Multiple lines of evidence suggest that the reduction in endothelium-derived NO is particularly important in driving both systemic and local CV disturbances during HFpEF progression. In HFpEF patients, NO bioavailability is reduced compared with that in control subjects (154), possibly owing to eNOS uncoupling (148). Experimental data show that aged NO-deficient mice display hypertension (155, 156) and other features of clinical HFpEF (157), including systemic microvascular rarefaction (heart, skeletal muscle), LV hypertrophy, myocardial fibrosis, and exercise intolerance (90, 158, 159). In addition to its role in vasomotor tone and blood flow regulation, endothelium-derived NO activates cardiomyocyte cGMP and protein kinase G (PKG) to elicit antihypertrophic and antifibrotic actions during cardiac stress (160–162). Along these lines, lower myocardial PKG signaling was associated with larger and stiffer cardiomyocytes in HFpEF patients (163, 164). PKG can also modulate the stiffness of the giant cytoskeleton protein titin, which is responsible for early diastolic recoil and late diastolic distensibility of cardiomyocytes (150). In both rat and human samples, increased resting cardiomyocyte tension is attributable to hypophosphorylation of the N2B isoform of titin because of low PKG activity (163, 164). Given these preclinical and clinical observations, there have been efforts to target NO/cGMP/PKG signaling in HFpEF patients. Despite some positive results in smaller clinical trials (165, 166), therapies targeting this axis in HFpEF patients have reported mostly negative or neutral outcomes in larger clinical trials (167, 168). At a mechanistic level, this suggests that impaired NO signaling is a consequence of systemic inflammation and, therefore, attempts to increase NO bioavailability without also targeting inflammation may be insufficient to produce clinical improvement.

Recent findings suggest that integrated vascular, immune cell, and cardiomyocyte responses to multiple metabolic/hemodynamic perturbations may be of more importance in driving the HFpEF syndrome than the microvasculature alone. For example, a “two-hit” model of hypertension (induced by the NO synthase inhibitor l-NAME) and HFD-induced obesity/IR (169) was the first mouse model that largely recapitulated several aspects of the clinical HFpEF phenotype, whereas each intervention alone only elicited varying degrees of diastolic dysfunction and LV remodeling. Similarly, a multiple-hit model of chronic salt water, unilateral nephrectomy, and aldosterone infusion (SAUNA mice) elicited diastolic dysfunction, cardiac inflammation, and fibrosis (170). In SAUNA mice, macrophages recruited from bone marrow and spleen to the heart induced collagen deposition via TGF-β1 release and subsequent activation of fibroblasts and myofibroblasts. Notably, cardiac microvascular ECs were also activated and expressed TGF-β1 in SAUNA mice, thus indicating immune-vascular synergy in driving cardiac inflammation and collagen deposition.

### Large-artery stiffness in HFpEF.

Several studies have demonstrated that large-artery stiffening is present in HFpEF patients to levels beyond normal age-related changes, either at rest or during exertion (171–175). Notably, aortic stiffening directly increases afterload as peak blood pressure is increased to overcome the reduction in aortic compliance (176). In addition, stiffer arteries generate faster pulse waves, shifting the arrival of reflective waves from diastole to late systole, which increases afterload while decreasing diastolic perfusion pressures (176). Together, these changes stimulate LV remodeling (177) and accentuate diastolic dysfunction (178). Furthermore, ventricular-arterial coupling dynamics are worsened in HFpEF patients during exercise, with insufficient changes in arterial compliance and increases in wave reflection being strongly associated with elevations in LV filling pressure and depressed cardiac output reserve (175). Interestingly, a component of aortic stiffness and ventricular-arterial coupling dysfunction may be due to impaired endothelium-dependent vasodilation in HFpEF patients, as sodium nitrite improved resting and exercise arterial load in a small clinical study (175).

### The lymphatic system in HFpEF.

The lymphatic system is responsible for returning most of the fluid filtered through semipermeable capillaries into the interstitial space back to the central venous system (179). Excess interstitial fluid accumulation (edema) is a result of an imbalance between fluid efflux from the vasculature and clearance by the lymphatic system. Edema in the periphery, abdominal viscera, and lungs is a characteristic feature of advanced HF that drives symptoms and adverse outcomes (180). Several hemodynamic and cellular mechanisms contribute to tissue edema in HF and were recently described in detail elsewhere (180, 181). In summary, vasoconstriction, sodium reabsorption, and microvascular dysfunction (rarefaction/activation) increase central venous pressure (CVP) that drives excess filtration. As the lymphatic network is a low-pressure system, elevation in CVP also impairs lymphatic drainage back into the central venous system. Impaired hemodynamic responses during exercise, such as elevated right- and left-sided pressures and impaired right ventricular–pulmonary artery coupling, exacerbate fluid filtration/clearance mismatch to promote pulmonary congestion, as demonstrated in approximately half of stable HFpEF patients (182). Moreover, several features of the lymphatic system are dysfunctional in HF patients, including impaired lymph vessel integrity/compliance, lymph valve dysfunction, impaired lymphangiogenesis, and lymph vessel rarefaction (181). Notably, a recent study showed lymph vessel rarefaction in skin biopsies from HFpEF patients, with residual lymph vessels being dilated and exhibiting less expression of markers of lymphatic differentiation and function in comparison with age-matched control participants (183). These changes in lymph vessel number/morphology were associated with impaired peripheral lymphatic drainage in HFpEF patients. Taken together, therapeutic targeting of the lymphatic system may offer additional benefits to standard therapy in the treatment of HFpEF. Likewise, stimulating lymphangiogenesis via coadministration of recombinant VEGFC156S during chronic angiotensin II (Ang II) infusion in mice largely attenuated cardiac remodeling; reduced cardiac inflammation, fibrosis, and chronic elevations in blood pressure; and improved kidney function in comparison with animals receiving Ang II alone (184). It will be exciting to test VEGFC156S therapy in models more closely aligning with HFpEF.

### Current approaches to treat metabolic HFpEF.

Considering the preclinical modeling strategies in the previous section, therapies targeting both metabolic and vascular stress may prove more efficacious than single-target therapies. Along these lines, the SGLT2 inhibitor empagliflozin was shown to reduce plasma glucose as well as blood pressure in hypertensive patients with T2D (185). Physiologically, empagliflozin stimulates osmotic natriuresis and reduces blood volume in stable, chronic HF patients with T2D (183). Recent and exciting findings from the phase III clinical trial EMPEROR-PRESERVE demonstrated that empagliflozin reduces the risk of the composite endpoint of CVD death or hospitalization for HF by 21% in adults with HFpEF with or without diabetes compared with placebo-treated patients (187). Interestingly, empagliflozin attenuated many features of clinical HFpEF in a three-hit mouse model; this was attributed to lessening inflammasome activation by increasing β-hydroxybutyrate and improving of mitochondrial function (188). Thus, beneficial mechanisms beyond reductions in blood volume are likely to occur with empagliflozin therapy, which may include improvement in vascular function and attenuation of proinflammatory signaling in microvasculature (189).

### Targeting the VEGFA/VEGFR2 signaling axis in cardiometabolic disease.

VEGFA/VEGFR2 signaling, microvascular density, and tissue perfusion are markedly reduced in several organs in aged mice (190). A recent study demonstrated, using transgenic mice or gene therapy, that a modest VEGFA increase in aged mice to levels observed in younger mice was able to alleviate age-related declines in systemic metabolism, reduce inflammation, and extend lifespan (190). Since age-related changes in the microvasculature are accelerated in cardiometabolic diseases, therapeutic targeting of the VEGFA/VEGFR2 axis may abrogate several features of cardiometabolic diseases. However, to date, evidence supporting the efficacy of *VEGFA* gene transfer in patients with ischemic disease has been limited (191) because of suboptimal vector delivery (192). Use of modified mRNA overcomes these issues, and modified mRNA encoding VEGFA has shown promise in preclinical models of post-infarct remodeling (193, 194) and diabetic wound healing (195), as well as small clinical trials in patients with T2D (193). Notably, intradermal delivery of modified mRNA encoding VEGFA was well tolerated in T2D patients and led to local increases in VEGFA protein and skin blood flow for up to 14 days after delivery. Currently, a phase IIa clinical trial has shown that direct injection of AZD8601, a synthetic mRNA encoding VEGFA_165_, into the myocardium of HFrEF patients with modestly reduced EF (30%–50%) improves EF, reduces circulating N-terminal pro–brain natriuretic peptide (NT-proBNP), and improves other patient-reported outcomes (197, 198). The future holds promise for further development and use of this technology in patients with metabolic disease and HFpEF.

## Summary

The inability of AT to store excess nutrients in the form of TGs leads to local AT inflammation and ectopic fat deposition in other metabolic tissues. Fat deposition in non-adipose tissue is strongly associated with IR and proinflammatory signaling, both of which impair tissue clearance of circulating TG-rich lipoproteins and result in a state of systemic inflammation. Together, IR and systemic inflammation enhance CVD risk and the incidence of HFpEF. Deeper understanding of the vasculature’s complex bidirectional role in the pathophysiology of atherosclerosis and cardiometabolic disease will reveal new opportunities and insights into whether EC dysfunction is a cause or a consequence of the disease pathogenesis. Importantly, treating underlying metabolic/inflammatory pathways in addition to improving vascular function may be the most efficacious treatment strategy to quell the progression of cardiometabolic disease.

## Version 1 03/15/2022

Electronic publication
